# Supplementary material for: ‘The community lives on sleeping medication and antidepressant[s]….’: Health care workers’ experiences of mental health service provision in rural South Africa
Source: PLOS Ment Health. 2025 Nov 13;2(11):e0000350. doi: 10.1371/journal.pmen.0000350 (PMC12798552; doi:10.1371/journal.pmen.0000350)
Supplement: S1 Text — (DOCX) [file pmen.0000350.s001.docx]

**Participant Demographic Questionnaire**

**Healthcare Provider**

Name and Surname:

Pseudonym (nickname):

Age:

Gender (M/F):

First language:

Level of education:

Occupation:

How long have you held this occupation status:
