# Supplementary material for: ‘The community lives on sleeping medication and antidepressant[s]….’: Health care workers’ experiences of mental health service provision in rural South Africa
Source: PLOS Ment Health. 2025 Nov 13;2(11):e0000350. doi: 10.1371/journal.pmen.0000350 (PMC12798552; doi:10.1371/journal.pmen.0000350)
Supplement: S2 Text — (DOCX) [file pmen.0000350.s002.docx]

**Qualitative Interview Schedule – Healthcare Provider**

1. Please tell me about yourself and your work responsibilities at this facility.
2. How long have you been working as (job description given in Question 1) at this facility?
3. What is your understanding about the umbrella term “mental healthcare”?
4. If you think about the patients who use this facility, what are the main mental health issues that they bring to the facility?
5. How common are these issues, and how are they usually dealt with?
6. The next few questions focus specifically on your role in providing mental healthcare at this facility. Would you mind discussing them with me?
   1. How long has mental healthcare provision been part of your work responsibilities?
   2. Did your role in mental healthcare provision evolve in any way over the years? Please elaborate.
   3. Can you explain the different mental healthcare treatment modalities available to service users at this facility?
   4. Who else is responsible for the provision of mental healthcare at this facility? Can you explain why you say so?
   5. What are your experiences with regards mental healthcare provision at this facility?
   6. What are the factors that make it easy or difficult for you to provide mental healthcare treatment here? Please elaborate why you say this?
   7. What do you think needs to change to improve the quality of mental health care provision at this facility, if any? Please explain your answer to me.
   8. What are the factors that are already in place that enhances the quality treatment mental healthcare at this facility? Please elaborate what makes you say so?
7. Are there any factors outside/away from this facility that impact your ability to provide mental healthcare to service users? Please elaborate on how it impacts mental healthcare provision here.
8. What does your colleagues or members of the community think about mental healthcare at the facility and in the area?
9. What would you wish tell key role players (e.g., the district manager or president) about the status of mental healthcare in your area or at this facility?
10. Please give me an example of a case where you felt you did well in helping a patient with a mental health problem.
11. Please give me a case where you felt you did not do well in helping a patient with a mental health problem
12. Is there anything else that you wish to tell me about mental health treatment provision that I must know?
