# Supplementary material for: ‘The community lives on sleeping medication and antidepressant[s]….’: Health care workers’ experiences of mental health service provision in rural South Africa
Source: PLOS Ment Health. 2025 Nov 13;2(11):e0000350. doi: 10.1371/journal.pmen.0000350 (PMC12798552; doi:10.1371/journal.pmen.0000350)
Supplement: Attachment — Submitted filename: Response to reviewers PLOS MH 110925.docx [file pmen.0000350.s004.docx]

11 September 2025

**TO:	The Editor**

**PLOS Mental Health**

**RE: Submission (ref no.: PMEN-D-25-00185) revised paper after peer review**

Dear editor and editorial team,

Thank you for considering our article for publication. We have reviewed and amended our paper after considering the feedback and inputs received from the editorial team and the peer reviewers. Please see detailed responses below. We first present the comment raised by the editor and reviewer(s) (*in italic font*). Then below it, we provide our response (**in bold font**) for the reader’s convenience.

**Responses to revisions requested**

Journal requirements:

*1. Please send a completed 'Competing Interests' statement, including any COIs declared by your co-authors. If you have no competing interests to declare, please state "The authors have declared that no competing interests exist". Otherwise please declare all competing interests beginning with the statement "I have read the journal's policy and the authors of this manuscript have the following competing interests:"*

**A competing interest section has been included in the manuscript.**

*2. We noticed that you used “unpublished data” in the manuscript. We do not allow these references, as the PLOS data access policy requires that all data be either published with the manuscript or made available in a publicly accessible database. Please amend the supplementary material to include the referenced data or remove the references.*

**We have removed this phrase and referral.**

*3. In the online submission form, you indicated that “All data are stored on the password protected computer and in locked cabinets of the PI. The datasets used and/or analyzed during the current study are available from the corresponding author on reasonable request.”.*

*All PLOS journals now require all data underlying the findings described in their manuscript to be freely available to other researchers, either 
1. In a public repository, 
2. Within the manuscript itself, or 
3. Uploaded as supplementary information.*

**Availability of data and materials section have been updated:**

**“All data underlying the findings described in the manuscript are within the manuscript itself.”**

*This policy applies to all data except where public deposition would breach compliance with the protocol approved by your research ethics board. If your data cannot be made publicly available for ethical or legal reasons (e.g., public availability would compromise patient privacy), please explain your reasons by return email and your exemption request will be escalated to the editor for approval. Your exemption request will be handled independently and will not hold up the peer review process, but will need to be resolved should your manuscript be accepted for publication. One of the Editorial team will then be in touch if there are any issues.*

**N/A**

*If the reviewer comments include a recommendation to cite specific previously published works, please review and evaluate these publications to determine whether they are relevant and should be cited. There is no requirement to cite these works unless the editor has indicated otherwise.*

**A number of relevant previously published work has been integrated into the revised manuscript in line with suggestions made by the editorial team and peer reviewers, as well as to expand our paper as requested. Citations and reference list has been updated accordingly.**

*Please review your reference list to ensure that it is complete and correct. If you have cited papers that have been retracted, please include the rationale for doing so in the manuscript text, or remove these references and replace them with relevant current references. Any changes to the reference list should be mentioned in the rebuttal letter that accompanies your revised manuscript. If you need to cite a retracted article, indicate the article’s retracted status in the References list and also include a citation and full reference for the retraction notice.*

**Reference list has been updated and checked.**

Additional Editor Comments (if provided):

*This is a timely and important paper, especially given the growing global momentum to integrate mental health into primary healthcare. The authors effectively highlight the perspectives of frontline health workers—the key drivers of these initiatives—offering valuable insights for policymakers, researchers, and program implementers. Their work provides critical food for thought on how best to operationalize this integration.*

**Thank you! We hope it can make a meaningful contribution to literature and the journal.**

Besides the comments from the reviewers here are my comments:

*1. The method section needs to be stronger. Can the authors provide more information on data analysis process. Was the code development/coding process done only by one person i.e. the first author? Who conducted these interviews?*

**The Research Methods section has been updated in attempt to make it stronger and more informative to the reader.**

*2. Getting more information of the participant would provide more richer perspective to the result section. Besides what you have in table 1, did you collect data on things like if they received any mental health training? If yes, please add*

**Table 1 has been updated to give extra information about the participants and also linked in our discussion section to add to the following comment of reviewer #2:** *What is the training of the current professionals you interviewed, and what is the gap? This will help the reader understand the context and the gap in services*

*3. My understanding is that the first author was a part of the system, as a psychologist. Can the authors add issues of personal biases, positionality as you conducted the research as well as during analysis and writing phase. How was the author’s own experience being a provider in the study area impact this paper?*

**A section on Positionality of the authors have been included in the Research Methods section.**

*4. As a stigma researcher, it was interesting to read about Theme 3- esp. subtheme 3 and 4 which fits well on how we define provider’s stigma. (Kohrt et al 2021 -* [*https://doi.org/10.1016/j.socscimed.2020.112852*](https://doi.org/10.1016/j.socscimed.2020.112852)*) The challenges faced by the health workers as well as the environment the authors explains how these forms of stigma is manifested. Can the authors expand more on this?*

**The suggested literature has been included in our paper. Thank you for pointing out.**

*5. It was also interested to read how health workers are relocating because of the challenges they are facing. This is exactly the opposite of what task sharing is trying to achieve – i.e. reducing treatment gap in the absence of health workers. Can the authors add more of this in the discussion section.*

**The discussion has been expanded:**

**Our findings resonate with our earlier work [13] which suggests that some staff who feel they are under enormous pressure may be discouraged to continue their services in MH care. Some staff, we found, have actually relocated to other facilities or departments to avoid experiencing such discomfort. Fewer health care workers willing to work in MH likely leads to higher care burden to the remaining few, which inevitably diminishes the quality of access to and provision of MH care in the area.**

*6. Overall, I feel the challenges authors describe here and the critical questions they ask applies to other settings too, esp. low-resource settings where the ultimate burden is falling upon health workers who are already over stretched. So I would suggest the author to consider expanding the scope of this paper – bringing in global perspectives and how the findings here imply to other similar places.*

**Manuscript has been updated to include reference and discussion of MH in low resource settings and low- and middle-income countries.**


[Note: HTML markup is below. Please do not edit.]

Reviewers' comments:

*Reviewer's Responses to Questions*

***Comments to the Author*** *1. Does this manuscript meet PLOS Mental Health’s* [*publication criteria*](https://track.editorialmanager.com/CL0/https:%2F%2Fjournals.plos.org%2Fmentalhealth%2Fs%2Fcriteria-for-publication/1/010f0197f43109ff-3957863c-5799-4bad-9ab8-ade23565922e-000000/65K1wi7Iv1CcD7mq5NC5ZXe-AKGnx8k05uK8ydfDp5w=219)*? Is the manuscript technically sound, and do the data support the conclusions? The manuscript must describe methodologically and ethically rigorous research with conclusions that are appropriately drawn based on the data presented.*

*Reviewer #1: Partly*

*Reviewer #2: Yes*

*2. Has the statistical analysis been performed appropriately and rigorously?*

*Reviewer #1: N/A*

*Reviewer #2: N/A*

*3. Have the authors made all data underlying the findings in their manuscript fully available (please refer to the Data Availability Statement at the start of the manuscript PDF file)?

The* [*PLOS Data policy*](https://track.editorialmanager.com/CL0/https:%2F%2Fjournals.plos.org%2Fmentalhealth%2Fs%2Fdata-availability/1/010f0197f43109ff-3957863c-5799-4bad-9ab8-ade23565922e-000000/FD-zzsgtRhQttQsaULz-umksHKNvhz-Up9IPYfJd0po=219) *requires authors to make all data underlying the findings described in their manuscript fully available without restriction, with rare exception. The data should be provided as part of the manuscript or its supporting information, or deposited to a public repository. For example, in addition to summary statistics, the data points behind means, medians and variance measures should be available. If there are restrictions on publicly sharing data—e.g. participant privacy or use of data from a third party—those must be specified.*

*Reviewer #1: Yes*

*Reviewer #2: Yes*

*4. Is the manuscript presented in an intelligible fashion and written in standard English?

PLOS Mental Health does not copyedit accepted manuscripts, so the language in submitted articles must be clear, correct, and unambiguous. Any typographical or grammatical errors should be corrected at revision, so please note any specific errors here.*

*Reviewer #1: Yes*

*Reviewer #2: Yes*

5. Review Comments to the Author

Please use the space provided to explain your answers to the questions above. You may also include additional comments for the author, including concerns about dual publication, research ethics, or publication ethics. (Please upload your review as an attachment if it exceeds 20,000 characters)

Reviewer #1:

This is a valuable and timely qualitative study exploring frontline health care workers’ experiences with mental health (MH) service provision in a rural, under-resourced region of South Africa. The manuscript offers rich narratives that shed light on structural challenges, resource limitations, and adaptations made by generalist providers working in the absence of adequate specialist support.

**Thank you!**

I have a few minor comments:

*1. Methodological Transparency: The abstract suggests qualitative methodology but lacks detail. Recommend elaborating on sampling methods, coding processes, reflexivity, and how trustworthiness (e.g., triangulation, audit trail) was ensured.*

**The methodology section has been expanded to provide further, deeper detail and transparency around methods followed to conduct the research.**

*2. Analytical Rigor: Clarify whether thematic saturation was reached, whether coding was independently verified, and if software (e.g., NVivo) or established procedures were used.*

**The Participant and Data Collection section mentions that it was deemed that data saturation was reached. The amended Data Analysis section describes the coding and analysis processes and that ATLAS.ti version 22 was used to organise the data.**

*3. Literature Integration: While the study references rural mental health literature, expanding the discussion to include broader qualitative analyses of psychotropic overuse and task-shifting in LMICs would enhance generalizability.*

**Literature on LMICs, and task-shifting have been incorporated into the Introduction in the Discussion.**

*4. Policy and Practice Implications: The manuscript would benefit from more concrete recommendations. For example, how can deprescribing frameworks, psychosocial supports, or community outreach models be practically piloted in similar contexts?*

**Recommendations have been added and restructured in the Conclusion and Recommendations section.**

Reviewer #2:

Abstract
*Health workers are burdened with high volumes of work in this part of the country. -repetitive in the previous sentence*

**Repetition has been removed in abstract.**

Introduction –

*would benefit from understanding the statistics or prevalence of mental health in SA.*

**Prevalence included in introduction.**

What are the policies related to mental health from the national department of health perspective?

**The introduction has been expanded to include literature of the National Department of Health’s National Mental Health Policy Framework and Strategic Plan (NMHPFSP) 2013-2020.**

*What services are supposed to be offered at the secondary and primary health care levels for mental health?*

**The introduction has been expanded to include literature of the National Department of Health’s National Mental Health Policy Framework and Strategic Plan (NMHPFSP) 2013-2020 on services expected by 2020.**

*This would create a better background for the reader*

**Thank you for your inputs.**

*Clarify if you are referring to acute psychological intervention or holistic services which include rehabilitation and integration. This will help the reader contextualise the student*

**Holistic mental health care specified in introduction.**

Methods
*How is this submission different from any other submission on the larger project that investigated aspects of access to and provision of public MH services? Given that mixed methods have to integrate data when reporting on the results.*

**Study design section edited:**

**“The presented work is the first to exclusively report qualitatively on the experiences of a group of frontline health workers that provide MH care in the area.”**

Research setting – needs to be explained as this is a international journal.

**Study setting has been explained to better contextualise the research setting.**

Data analysis
*Transcribed narrative – does this refer to transcript- this may confuse the reader*
**This has been rephrased to: “the narratives that were transcribed”…**

Results
*Valium- please change the pseudonym as this is a drug used- may confuse the reader*

**Pseudonym has been changed to “Participant X” and footnote inserted to provide clarity and context. Pseudonym has been changed throughout text for uniformity.**

*Quote not clear. Person states that they encounter persons who are suicidal, but quote says there wouldn’t be such high incidences of people committing suicide
Niesie reported that they also encounter persons who become suicidal because they are
236 depressed: ‘There wouldn’t be such high incidences of people committing suicide … when
237 they feel very depressed or low ….’*

**Quote has been updated.**

Discussion
*Task shifting is a reality in low and middle-income countries at the secondary and tertiary levels. What is the training of the current professionals you interviewed, and what is the gap? This will help the reader understand the context and the gap in services*

**We have updated Table 1.**

**We also added the following in the discussion section to illustrate a gap:**

**This is so, especially in contexts like the area investigated where, as shown by our sample, not all health care workers are adequately trained in MH and very limited continued professional development or education has occurred around MH over the past years [42], as envisaged by the NMHPFSP 2013-2020. It further seems that ironically, refresher courses were attended only by higher level clinicians like medical officers and not primary health nurses, who are the health workers who see the majority of patients [42].**

6. PLOS authors have the option to publish the peer review history of their article ([what does this mean?](https://track.editorialmanager.com/CL0/https:%2F%2Fjournals.plos.org%2Fmentalthealth%2Fs%2Feditorial-and-peer-review-process%23loc-peer-review-history/1/010f0197f43109ff-3957863c-5799-4bad-9ab8-ade23565922e-000000/fglealO4pnQqq7hFzv4832c2cf5BmL-scMtJYMzZqaU=219)). If published, this will include your full peer review and any attached files.


**Do you want your identity to be public for this peer review?** If you choose “no”, your identity will remain anonymous but your review may still be made public.


For information about this choice, including consent withdrawal, please see our [Privacy Policy](https://track.editorialmanager.com/CL0/https:%2F%2Fwww.plos.org%2Fprivacy-policy/1/010f0197f43109ff-3957863c-5799-4bad-9ab8-ade23565922e-000000/R5HT4L0rtlPO6U-GIBoeGUVYZtCUQ_Bgpez1Mv2sm_g=219).

Reviewer #1: No

Reviewer #2: **Yes:** Deshini Naidoo

We trust that you will find our responses above in order and appropriate for re-submission. Please do not hesitate to enquire if further information or inputs from our side are required.

Looking forward to response for your side.

Kindest regards,

Divan Rall
